# A novel silent speech recognition approach based on parallel inception convolutional neural network and Mel frequency spectral coefficient

**DOI:** 10.3389/fnbot.2022.971446

**Published:** 2022-09-02

**Authors:** Jinghan Wu, Yakun Zhang, Liang Xie, Ye Yan, Xu Zhang, Shuang Liu, Xingwei An, Erwei Yin, Dong Ming

**Affiliations:** ^1^Academy of Medical Engineering and Translational Medicine, Tianjin University, Tianjin, China; ^2^Tianjin Artificial Intelligence Innovation Center (TAIIC), Tianjin, China; ^3^Defense Innovation Institute, Academy of Military Sciences (AMS), Beijing, China; ^4^Department of Electronic Science and Technology, University of Science and Technology of China, Hefei, China

**Keywords:** surface electromyography (sEMG), silent speech recognition, Mel frequency spectral coefficient, convolutional neural network, subject-based transfer learning

## Abstract

Silent speech recognition breaks the limitations of automatic speech recognition when acoustic signals cannot be produced or captured clearly, but still has a long way to go before being ready for any real-life applications. To address this issue, we propose a novel silent speech recognition framework based on surface electromyography (sEMG) signals. In our approach, a new deep learning architecture Parallel Inception Convolutional Neural Network (PICNN) is proposed and implemented in our silent speech recognition system, with six inception modules processing six channels of sEMG data, separately and simultaneously. Meanwhile, Mel Frequency Spectral Coefficients (MFSCs) are employed to extract speech-related sEMG features for the first time. We further design and generate a 100-class dataset containing daily life assistance demands for the elderly and disabled individuals. The experimental results obtained from 28 subjects confirm that our silent speech recognition method outperforms state-of-the-art machine learning algorithms and deep learning architectures, achieving the best recognition accuracy of 90.76%. With sEMG data collected from four new subjects, efficient steps of subject-based transfer learning are conducted to further improve the cross-subject recognition ability of the proposed model. Promising results prove that our sEMG-based silent speech recognition system could have high recognition accuracy and steady performance in practical applications.

## 1. Introduction

Automatic speech recognition (ASR) has a long history of research (Bahl et al., 1983; Hinton et al., 2012; Chu et al., 2020). By audio signal processing and modeling, speech contents can be transcribed into texts for various applications (Yu and Deng, 2016; Yang et al., 2021). Yet in particular cases, the audio signals cannot be clearly produced or captured. For example, ASR is improper for patients and the elderly with speech disorders like dysphonia, because the utterances cannot be pronounced and recorded clearly (Green et al., 2003). Furthermore, some emergency situations like the sudden loss of voice and speech impediment may require special speech interaction assistance as well (Jong and Phukpattaranont, 2019). To this end, silent speech recognition (SSR) is proposed to overcome the limitations of ASR under the above circumstances (Meltzner et al., 2018).

**Silent Speech Recognition:** With the absence of exploitable audio input, multiple biomedical modalities and signals have been implemented in the decoding of human intentions through SSR (Ji et al., 2018). Permanent magnet pellets, for example, are used in Hofe et al. (2013) to capture permanent-magnetic articulography (PMA) speech data as additional information for speech recognition. Similarly, electromagnetic articulograph (EMA) based SSR uses sensors adhered to the tongue and lip to record articulatory movements during speech (Kim et al., 2017). Ultrasound images of the lip region can be used as speech clues as they provide the real-time position of the tongue during speech, and sometimes they are combined with optical images (Hueber et al., 2008; Cai et al., 2011) to give better recognition performance. Electro-optical stomatography (EOS) adopts electrical sensors and optical sensors to record the complete trace of lip and tongue movements during speech (Stone and Birkholz, 2016). Although electromagnetic signals, ultrasound, and optical images are used for speech-related information decoding, they have some limitations regarding the data acquisition methods. For example, the use of electromagnetic sensors adhered to the tongue directly in the mouth may bring health concerns, and the ultrasound probe is not suitable for portable applications. Although EOS is proposed to extract speech information from the lip region especially, there are too many sensors placed in the mouth, affecting the normal speech process. From this aspect, surface electromyography (sEMG) signal can be captured easily from the surface of body skin (Englehart et al., 1999) and has been used in previous studies for speech recognition from about the 1980s (Morse et al., 1989), showing satisfactory performance over the time (Denby et al., 2010; Schultz et al., 2017).

**Surface EMG Feature Extraction:** sEMG is produced by muscle movements according to the electrical propagation of central and peripheral nerves (Chowdhury et al., 2013a). It is captured by surface electrodes, which are adhered to speech-related muscles and have very little influence on the subject during speech. While at the same time, it is non-stationary and difficult to be processed because of the electrophysiology principles and irregular motor unit discharges during muscle activities (Karlsson et al., 1999; Xie and Wang, 2006; Chowdhury et al., 2013b). As a result, different types of features and feature extraction methods have been adopted in sEMG signal processing. As summarized in Srisuwan et al. (2018) and Mendes Junior et al. (2020), time domain features such as Mean Absolute Value (MAV), Root Mean Square (RMS), and Variance (VAR) describe how sEMG signals vary temporally. They can be extracted in a simple but fast way and have been widely used in the study of sEMG signals (Hudgins et al., 1993; Englehart et al., 1999; Tkach et al., 2010; Samuel et al., 2018). On the other hand, frequency domain features are also used in the recognition and classification of sEMG signals, especially for the study of muscle fatigue (Phinyomark et al., 2012). However, in some cases they show relatively poor recognition results compared to using time-domain features (Srisuwan et al., 2018; Jong and Phukpattaranont, 2019; Mendes Junior et al., 2020). Furthermore, studies show better recognition performance by combining several types of features to form a new feature vector of the collected data (Atzori et al., 2016; Mendes Junior et al., 2020). Recently, spectral features such as Mel-Frequency Cepstral Coefficients (MFCC) (Meltzner et al., 2017; Kapur et al., 2018; Zhang et al., 2020) tend to be more popular in both acoustic speech recognition and sEMG-based silent speech recognition. They have relatively higher dimensions and can be used to extract more speech-related characteristics from the original signals.

**Surface EMG based Silent Speech Recognition:** Classification algorithm plays an important role in sEMG-based silent speech recognition. Statistical model Hidden Markov Model (HMM) is one of the most classical recognition methods for both automatic speech recognition and silent speech recognition, and has been widely implemented in relevant studies (Meltzner et al., 2011, 2017, 2018; Kubo et al., 2013). With the development of high-performance computers and data acquisition techniques, machine learning algorithms such as the basic Feedforward Neural Networks (Jong and Phukpattaranont, 2019), Linear Discriminant Analysis (LDA) (Liu et al., 2020), Bayes network (Dobrucki et al., 2016), Random Forests (RF) (Rameau, 2020; Zhang et al., 2020), and Support Vector Machine (SVM) (Rameau, 2020) have been used for silent speech recognition. Recently, deep learning has achieved great success in pattern recognition tasks, among which Convolutional Neural Network (CNN) shows outstanding performance not only in image classification but also in speech recognition (Liu et al., 2018; Xiong et al., 2018; Rashno et al., 2019). Successful implementations of CNN in sEMG-based silent speech recognition are reported as well. For example, Kapur et al. (2018) employed 1-dimensional CNN to process MFCC features extracted from sEMG data and used a deep structure to predict the word label in the output. Under laboratory circumstances, it can be easy to collect high-quality data for machine learning algorithms, which is not consistent with the real application situation outside. The research of robust recognition algorithms and transfer learning strategies is necessary when it is difficult to acquire enough data for model training and cross-subject application in practice.

The combination of spectral features and machine learning algorithms has been widely accepted in sEMG-based silent speech recognition, yet it is not guaranteed to have satisfactory recognition accuracy because of the complex frequency components and the noise mixed in sEMG signals during data acquisition. What's more, physiological signals rely heavily on the physiological conditions of subjects, which brings great challenges for cross-subject recognition. More effective methods are required both in feature extraction and recognition, especially for real-life applications. From this aspect, we carried out this research on the facial sEMG-based silent speech recognition approach and provided a robust interaction system for different users. Through the experiments of data collected from healthy subjects, and the research of transfer learning for cross-subject scenery, we constructed our sEMG-based silent speech recognition system at this step, and we were prepared for further practical applications. Our main contributions in this paper include:

- Proposing a novel deep learning architecture named Parallel Inception Convolutional Neural Network (PICNN). This parallel processing architecture is designed to further extract and recognize spatial features from the spectrum of sEMG signals. It is proven to have the best recognition performance in offline evaluation and robustness in cross-subject experiments, showing great potential for future implementation in daily life assistance.
- Design and generate a 100-class Chinese phrase corpus with 28 healthy subjects who participated in sEMG data collection, containing short demands and utterances in medical care and living assistance for various users. The corpus simulates basic application circumstances for the proposed silent speech recognition system and provides a research platform for cross-subject silent speech recognition research in this paper.
- Implement a subject-based transfer learning strategy for cross-subject experiments and improve the recognition ability of the proposed PICNN model. By simple steps of fine-tuning with a limited amount of new data, pre-trained models can be used for new subjects with steady performance. It is an essential foundation for practical applications of sEMG-based silent speech recognition systems.

In the following part of this paper, the construction and pre-processing procedures of the sEMG speech corpus are described in detail in Section 2. Section 3 introduces the newly developed PICNN model. Section 4 presents experimental details, including feature extraction methods and baseline models used in following experiments. Results of our research are delivered in Sections 5, 6 gives some further discussions. Section 7 makes a conclusion of this work and proposes outlook in the future.

## 2. Cross-subject sEMG speech dataset

### 2.1. Corpus design

Based on the five levels of Maslow's Hierarchy of Needs (Maslow, 1943), we design 100 classes of daily assistance demands in Chinese for a wide range of users, especially the elderly and patients with speech disorders. Five categories of demands, including physiology, safety, social interaction, self-respect, and fulfillment, as well as entertainment requirements are considered in the design of demand contents to cover most of the interaction needs and meet as many potential requirements as possible in daily life. There are three to five different Chinese characters in each demand. Specifically, 18 demands contain three characters, 48 demands contain four characters and the remaining 34 demands contain five characters. There are some examples listed in Table 1 with phonetic Mandarin transcriptions of their pronunciation.

**Table 1:** Examples of the utterances in corpus.

**Category**	**Label**	**Demand**	**Phonetic transcription in mandarin**	**English translation**
Physiology	8	我要上厕所	wo3yao4shang4ce4suo3	I'm going to the toilet
Safety	28	紧急呼救	jin3ji2hu1jiu4	Emergency
Social Interaction	65	我要发短信	wo3yao4fa1duan3xin4	I want to send a text message
Self-respect and Fulfillment	77	我能行的	wo3neng2xing2de5	I can do it
Entertainment	89	我要看电视	wo3yao4kan4dian4shi4	I want to watch TV

### 2.2. sEMG signal acquisition

**Figure 1:**
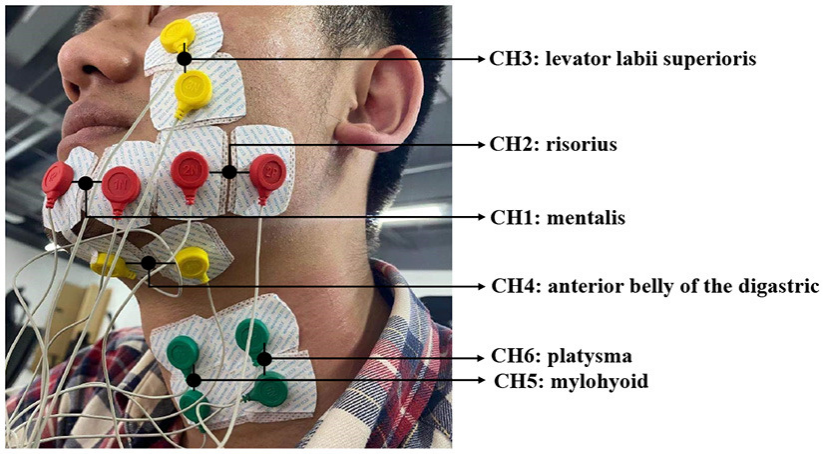
Positions of paired electrodes adhered on subject's face and neck for data acquisition.

The sEMG data is captured using surface electrodes from six muscles on the subject's face and neck, i.e., mentalis, risorius, levator labii superioris, anterior belly of the digastric, mylohyoid, and platysma (indicated as CH1 to CH6), which are all related to the speech process. The device we use for sEMG signal acquisition is NSW308M bipolar EMG system produced by Neuracle Technology. Six channels of sEMG data collection electrodes are placed on the surface of corresponding muscles, as shown in Figure 1 below. An additional reference electrode is placed upon the collarbone in order to record the potential difference of the human body as a baseline.

**Figure 2:**
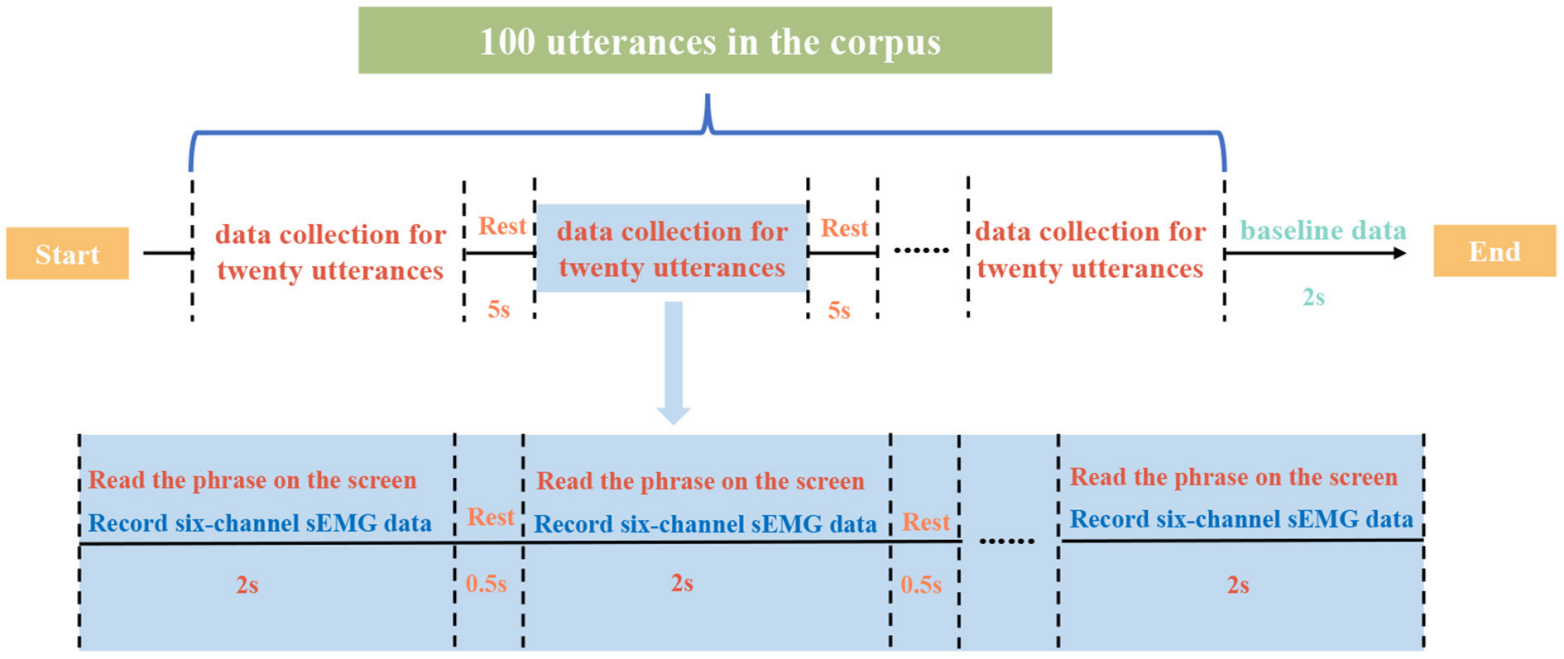
Data collection experiment timeline for one session.

The sEMG data in the dataset is collected from 28 normal subjects (nineteen male and nine female subjects, aged from 23 to 32, with a mean age of 26.21), with Mandarin as their mother language. During the data collection experiment, each subject is required to utter the Chinese phrases in a low voice. When the phrase is displayed on the screen and sustained for 2 s, we record the real-time sEMG signals from corresponding muscles simultaneously. A timeline for the data collection process is shown in Figure 2. Some demands shall be uttered quicker if there are more characters while some can be uttered slowly if there are fewer words. All the 100 phrases in the corpus are read once in one session and each subject repeats 10 sessions during the whole data collection experiment. Additionally, a non-content class of sEMG data is recorded at the end of each session (indicated as baseline data in Figure 2) so there are 101 classes of sEMG data in one session. For the cross-subject sEMG speech dataset used in this research, there are 280 sessions of sEMG data collected from 28 subjects, including 28,078 pieces of valid sEMG data (we delete corresponding sEMG data when the subject makes a mistake uttering the phrase) each 2-s long. Data acquisition experiments are approved by the Institutional Review Board of Tianjin University (TJUE-2021-138). The informed consent form is read and signed by each subject before experimental procedures.

### 2.3. Signal pre-processing

Raw sEMG data is recorded at the sampling rate of 1,000 Hz. Although they have undergone a series of processing steps such as filtering and de-noising by the software built in the acquisition system, there are still several types of interference such as DC offset, 50 Hz power frequency noise, and peak amplitude mixed in the sEMG data. These noises will result in a low signal-to-noise ratio (SNR), therefore signal pre-processing is required. On the other hand, we plan to release our corpus and dataset for more research in sEMG-based silent speech recognition in the near future. For convenience of use, it is important to conduct necessary pre-processing steps to improve our data quality. To be specific, a Butterworth notch filter for 50 Hz power frequency noise removal is first applied to the raw data. The frequency range of effective sEMG signals is mainly distributed at 10–400 Hz (Lyu et al., 2014), thus a Butterworth band-pass filter of 10–400 Hz is then implemented.

## 3. Parallel inception convolutional neural network

### 3.1. Fine-grained feature combination

**Figure 3:**
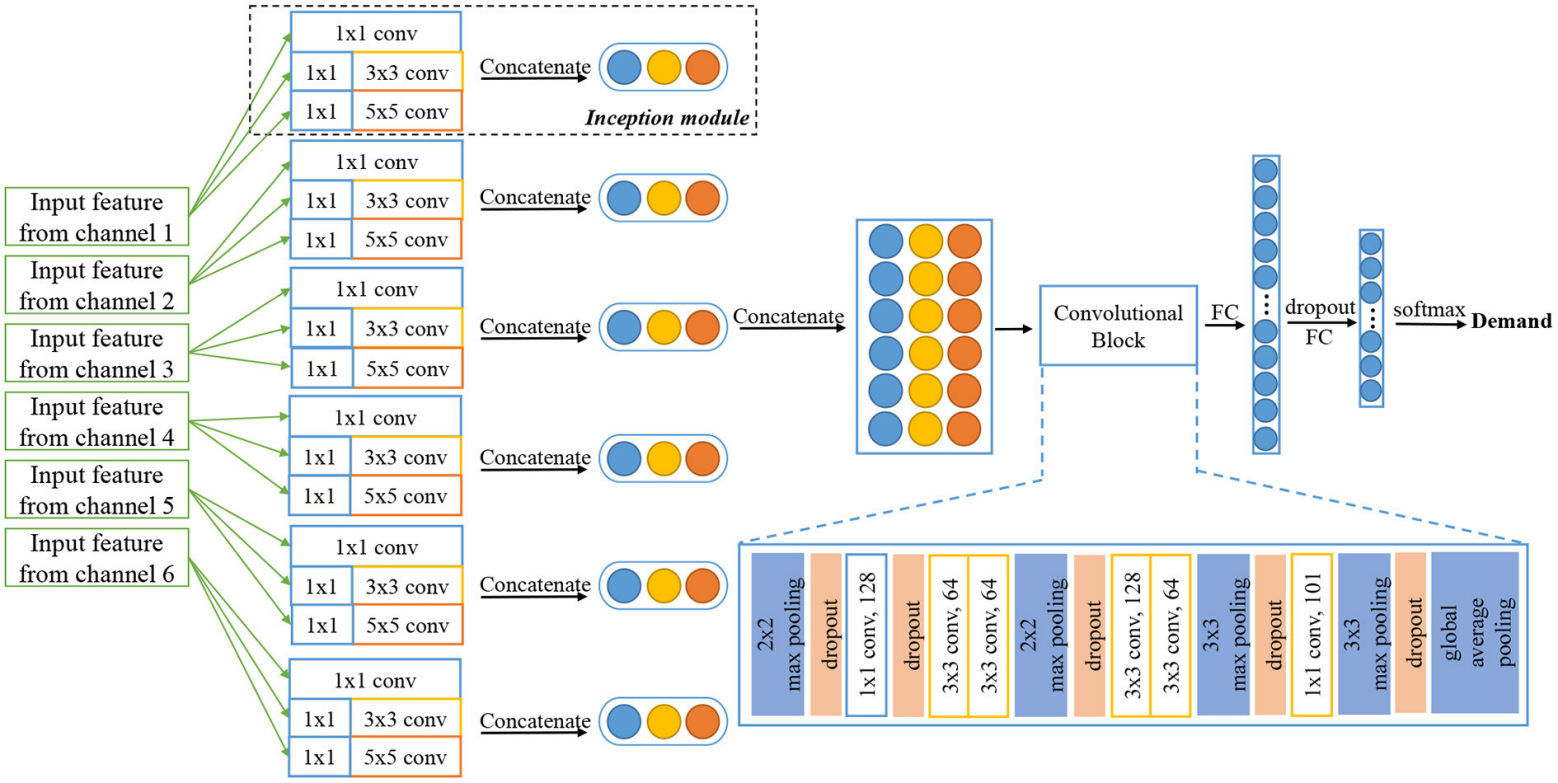
Architecture of proposed PICNN model.

In conventional research, sEMG data collected from different muscles tend to be considered as a whole and fed into the following recognition system. However, innovated by the original framework of Inception architecture and GoogLeNet (Szegedy et al., 2015), its outstanding upgrades (Szegedy et al., 2016, 2017) and Xception architecture (Chollet, 2017), we find it may be efficient to process sEMG signals in each channel separately to obtain the unique spatial features among six channels and regional features within each channel. The design of parallel architecture is to better extract muscle activity patterns from different positions on the face and neck, and keep their particular characteristics during model training and recognition. From this aspect, we propose our sEMG-based silent speech recognition model Parallel Inception Convolutional Neural Network (PICNN) in this paper. The newly proposed PICNN model adopts parallel convolutional architecture, indicated as the Inception module, in the front end of the model to process the input from each data channel. As shown in Figure 3, six inception modules are used to extract fine-grained features from each channel, respectively, while keeping the spatial correlations of six channels as another latent information for recognition. An inception module is consisted of three sizes of convolutional filters (i.e., 1 × 1, 3 × 3, 5 × 5), with 32 feature maps for each size of them. Input sEMG data are filtered by three types of filters respectively and a total of 96 feature maps can be obtained by concatenating the three types of feature outputs. In practice, we adopt the architecture of the inception module with dimension reductions, as raised in Szegedy et al. (2015). A Max pooling layer is added after concatenation to further reduce the feature dimension. For two-dimensional spectral feature input, we use 2D convolution layers and for one-dimensional features like time domain features, we merely change to use the 1D convolution layer but keep the whole architecture and filter size the same.

### 3.2. PICNN architecture

Surface EMG data from six channels are processed by six Inception modules described above simultaneously, and the output feature maps are concatenated one by one to form a feature vector with a total number of 576. In parallel processing, parameters of convolution kernels used in each channel are updated after training and error propagation, which means we have extracted regional characteristics from six channels of sEMG data collected in the previous procedure. After that, a common convolutional neural network structure is adopted for feature processing and Softmax is used as the final activation function for classification. As shown on the right side of Figure 3, the remaining part of the proposed PICNN has a total of six convolution layers, four max pooling layers and a global max pooling layer to form a deep convolutional neural network, indicated as Convolution Block in the Figure 3. The number of hidden nodes in the last fully connected layer is 101 in this study, according to the number of classes in the speech corpus we collected. Besides, each convolution layer is followed by the Batch Normalization layer to avoid fluctuations of model parameters during training and testing (Ioffe and Szegedy, 2015). Dropout is also added to each max pooling layer to resist overfitting, and 25% of the neurons will be randomly dropped in this process. Leaky Rectified Linear Unit (Leaky ReLU) is used as an activation function in the whole PICNN architecture.

## 4. Experiment

### 4.1. Feature extraction methods

Time domain features are widely used in sEMG signal processing and sEMG-based silent speech recognition (Wand et al., 2014; Srisuwan et al., 2018), so we use some of them in our research as well. We omit the first 250 ms of sEMG data as the reaction period of each subject, and then a sliding time window with a fixed length of 200 ms is used here. It moves 50 ms forward each step so the total number of time windows is 32 for each channel of sEMG data. The total number of sliding windows for each utterance is always the same because the data length is 2 s, no matter how long the utterance is. Considering the 1,000 Hz sampling rate, there are 2,000 sampling data points in each piece of sEMG data from each channel. The window length of 200 ms and hop size of 50 ms were determined by a pre-research based on previous studies (Smith et al., 2011; Mendes Junior et al., 2020). We selected three window length values of 200, 500, and 1,000 ms, combined with three overlap rates of 0.25, 0.5, and 0.75 for the sliding time windows. Paired experiments were conducted to compare the recognition accuracy, and we observed that 200 ms of window length with an overlap of 0.75 (which indicated the hop size of 50 ms) gave the best recognition performance.

In this paper, four time domain features are selected and tested, those being Mean Absolute Value (MAV), Variance (VAR), Root Mean Square (RMS), and Mean Waveform Length (MWL). Mean Absolute Value (MAV) is a common feature used in sEMG signal pattern recognition and related applications (Oskoei and Hu, 2007; Phinyomark et al., 2009). It is used to measure the overall muscle activities and represent muscle movements during speech. For a time series x(i), it is defined as:


(1) $$\begin{array}{r} MAV_{k}=\frac{1}{W}\overset{W}{\underset{i=1}{\sum }}|x\left(i\right)| \end{array}$$

where *k* is the *k*th time window (the same definition is used below) and *W* is the length of sliding time window (i.e., 200 sample points according to sampling rate). These variables are kept the same in the following feature extraction methods.

Variance (VAR) reflects the deviation between signal amplitude and its mean value. It also detects clustering characteristics of certain muscle activity from corresponding sEMG signals. We use population variance here and the mathematical definition of VAR is:


(2) $$\begin{array}{r} VAR_{k}=\frac{1}{W}\overset{W}{\underset{i=1}{\sum }}{\left(x\left(i\right)-\frac{1}{W}\overset{W}{\underset{i=1}{\sum }}x\left(i\right)\right)}^{2} \end{array}$$

Root Mean Square (RMS) relates to the average power of the movement. RMS values of different signal channels can reflect the contribution of the muscles in different positions when speaking. RMS can be calculated by:


(3) $$\begin{array}{r} RMS_{k}=\sqrt{\frac{1}{W}\overset{W}{\underset{i=1}{\sum }}x{\left(i\right)}^{2}} \end{array}$$

Mean Waveform Length (MWL) is the overall measure of muscle activities, and it also indicates the complexity and persistence of the sEMG signals. MWL can be defined as:


(4) $$\begin{array}{r} MWL_{k}=\frac{1}{W}\overset{W-1}{\underset{i=1}{\sum }}|x\left(i+1\right)-x\left(i\right)| \end{array}$$

We use each of them as the independent feature input in recognition experiments and then combine them to form a new feature vector named TD4. What's more, our previous research (Wu et al., 2021) indicates that the combination of both time and frequency domain features can have better recognition performance, so we add another two frequency domain features Mean Frequency (MNF) and Median Frequency (MDF) into the feature vector and combine them as TFD6. Mean Frequency (MNF) is the centroid frequency of the Power Spectral Density (PSD) obtained from sEMG signal. It is calculated as follows:


(5) $$\begin{array}{r} MNF_{k}=\frac{\sum_{i=1}^{W}f_{i}P_{i}}{\sum_{i=1}^{W}P_{i}} \end{array}$$

where *f* refers to the frequency and *P* is the PSD corresponding to *f*. Median Frequency (MDF) is the frequency that divides the PSD into two equal parts. It satisfies the following equation:


(6) $$\begin{array}{r} \overset{MDF}{\underset{i=1}{\sum }}P_{i}=\overset{\infty }{\underset{i=MDF}{\sum }}P_{i}=\frac{1}{2}\overset{\infty }{\underset{i=1}{\sum }}P_{i} \end{array}$$

Apart from time domain and frequency domain features, spectral features Short Time Fourier Transform (STFT), Mel-Frequency Cepstral Coefficient (MFCC), and log Mel frequency spectral coefficient (MFSC, also referred to as Mel-filterbanks in Zeghidour et al., 2018) are used in the feature extraction as well. STFT sets finite windows on the non-stationary signal and calculates the Fourier transform within the windowed signal. It can be obtained by:


(7) $$\begin{array}{r} STFT\left(t,f\right)=\int_{-\infty }^{\infty }x\left(\tau \right)h\left(\tau -t\right)e^{-j2\pi f\tau }d\tau \end{array}$$

where *h*(τ−*t*) is the window function, *f* and τ are modulation frequency and translation parameter, respectively. MFSC is used for sEMG signal feature extraction for the first time. The feature extraction method of MFSC is similar to MFCC, including pre-emphasis, framing and windowing, Fast Fourier Transform (FFT), Mel filter bank filtering, and logarithm in the end (Mohamed, 2014), while it omits the last step of Discrete Cosine Transform (DCT) in the feature extraction of MFCC (Zheng et al., 2001). Another convenient way to obtain MFSC is to perform a Short Time Fourier Transform (STFT) of the input signal and then take averages of triangular filters in the frequency domain, as defined in Zeghidour et al. (2018). We adopt these three features, i.e., STFT, MFCC, and MFSC to compare the recognition performance of spectral features and the above feature extraction methods.

In practice, we implement 36 Mel filters and 36 frames to form the MFSC features from each channel of sEMG signals. For MFCC features, we implement 36 frames and a frame of 12 Mel filters, first-order derivative, and second-order derivative of the Mel filters, to form a 36 × 36 dimensional feature matrix for each channel of data. The python package *librosa* (doi: 10.5281/zenodo.3955228) is used to obtain MFSC and MFCC features during this process. We set the length of the FFT window to 200 and the hop length to 50, respectively. For STFT features, we use the python package *signal* from the Python SciPy library.

### 4.2. Baseline models

According to the previous research in the sEMG based silent speech recognition area, we selected some commonly used machine learning algorithms and state-of-the-art deep learning architectures as baseline models to compare the recognition and classification ability. Three machine learning algorithms, i.e., Random Forest (RF), Linear Discriminant Analysis (LDA), and Support Vector Machine (SVM) are used here because of their successful implementations in sEMG pattern recognition studies. The deep learning architecture, Convolutional Neural Network (CNN) is chosen, which is one of the most basic but popular deep learning architectures in several deep learning fields such as computer vision and natural language processing. As our model is a more complex architecture of CNN, we would like to compare it with this basic model to see how the proposed PICNN outperforms conventional models in this research.

Particularly, CNN used in this paper is constructed upon a famous structure named VGGNet (Simonyan and Zisserman, 2014). We make some slight adjustments to the number of convolution layers and filter sizes of VGG16 to fit in our dataset. The number of feature maps obtained by each convolution layer is reduced by half and we also omit the last few of them in the original VGG16 architecture due to the data shape of input features.

**Figure 4:**
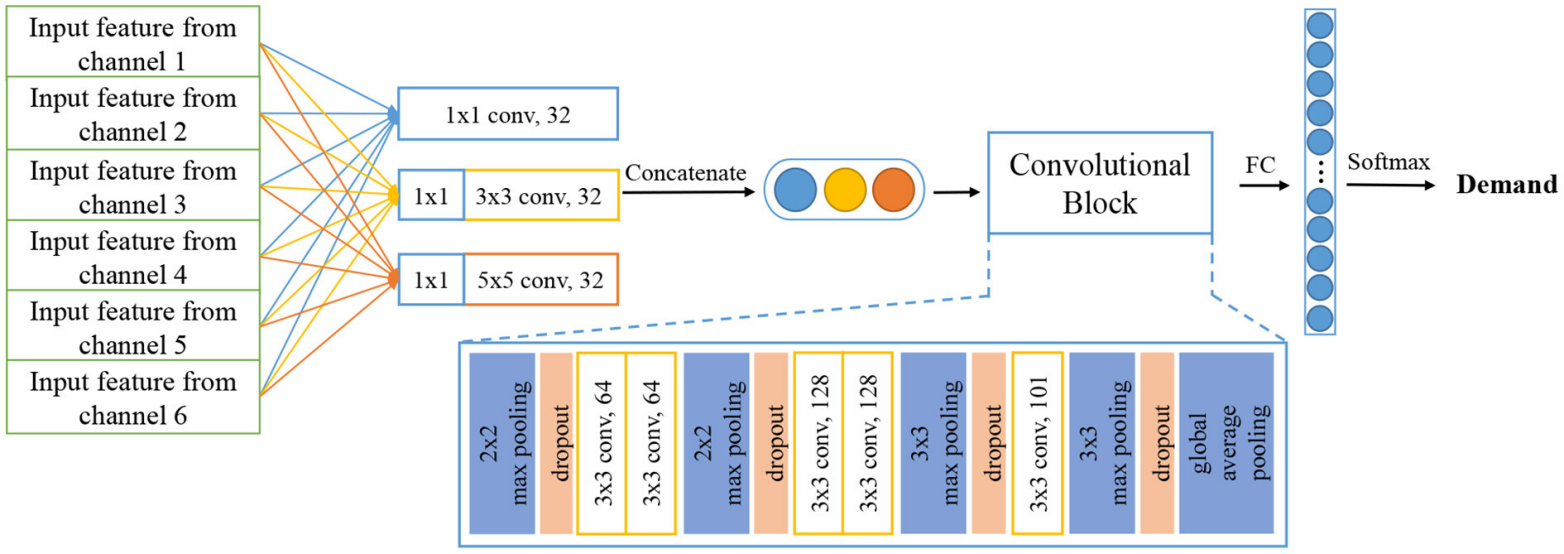
Structure of inception model used in this paper.

Further, we adopt another structure of CNN as the baseline model. It is Inception architecture, from which our model PICNN is innovated and further developed. Inception architecture is used to extract spatial information and regional features by grouped convolution layers with different kernel sizes. It originally appeared in GoogLeNet (Szegedy et al., 2015) for computer vision and image processing tasks, achieving outstanding performance as expected. We use this Inception module and construct a similar structure of GoogLeNet as a baseline model. Different from PICNN, sEMG features of six channels are first concatenated as a larger input and then fed into convolutional kernels with sizes of 1 × 1, 3 × 3, and 5 × 5, respectively. The feature maps obtained from each convolution layer will be further concatenated to form a feature vector and fed into a deep convolution block afterward. It is followed by a fully connected layer and Softmax to give a recognition result. To be conveniently referred to in the following experiments, we simply name this structure as Inception, as shown in Figure 4.

### 4.3. Surface EMG based silent speech recognition system and experiment settings

**Figure 5:**
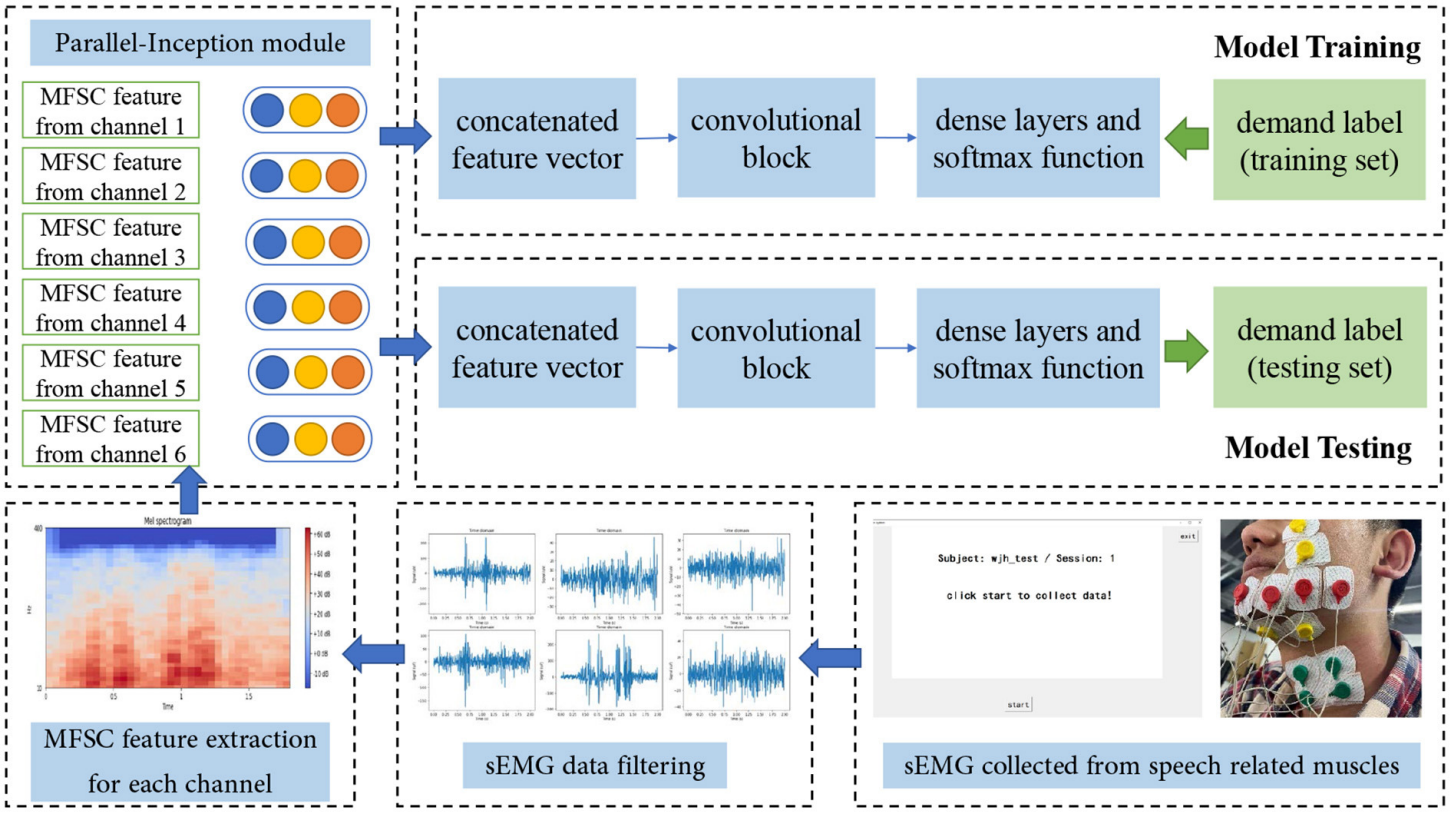
Diagram of proposed sEMG-based silent speech recognition system.

The diagram of our sEMG-based silent speech recognition system using the PICNN model is shown in Figure 5, and we take MFSC feature input as an example to show the whole data processing pipeline. sEMG data from the training set and corresponding labels are used in the offline training process, while the model parameters obtained from this step are used for model testing to further analyze the recognition and classification ability of our system. The whole dataset we obtained from 28 subjects is first shuffled randomly. After that, 80% of the data is used for model training and validation, and the rest 20% is used for testing. From this aspect, we focus on the cross-subject recognition ability of our model, rather than the recognition ability for each subject, respectively.

We carry out data collection programs and recognition experiments in Python. Relevant models and classification tools in the Python library scikit-learn are used in the realization and evaluation of three machine learning algorithms. For baseline models CNN and Inception, as well as our PICNN model, we code on our own with Tensorflow and Keras rather than using available model packages.

## 5. Results

We use the classification accuracy on the test set as the evaluation of model recognition ability. It is defined as follows:


(8) $$Accuracy=\frac{number\;of\;correctly\;predicted\;labels}{total\;number\;of\;labels}$$

Apart from it, the recognition rate for each class of Chinese phrase is also adopted to show the robustness of our model. We define the recognition rate as follows:


(9) $$Recognition\;rate=\frac{TruePositive}{TruePositive+FalseNegative}$$

which is the same definition as *recall* in classic classification tasks. We use these two evaluation metrics (i.e., classification accuracy and recognition rate) in the next recognition experiments.

### 5.1. Comparison of feature extraction methods and recognition models

The classification accuracy for each pair of feature extraction method and classifier is shown in Table 2. It was obvious that our PICNN model achieved the highest classification accuracy among these six recognition models using different feature extraction methods, far exceeding the machine learning algorithms LDA, RF, and SVM in this research. The classification accuracy reached 90.76% for MFSC features and the PICNN model, which indicated the best recognition performance of our sEMG-based silent speech recognition system. The results showed the effectiveness and promising recognition ability of our sEMG data acquisition system and recognition system, getting us prepared to invite the elderly and patients with speech disorders to participate in our research.

**Table 2:** Classification accuracy (%) for different feature extraction methods and classifiers.

**Classifier**	**Feature extraction method**								
	**MAV**	**VAR**	**RMS**	**MWL**	**TD4**	**TFD6**	**STFT**	**MFCC**	**MFSC**
LDA	19.11	16.65	18.71	19.36	25.04	24.12	16.15	12.96	7.34
RF	42.31	40.44	40.87	47.35	44.27	43.41	39.17	24.41	29.90
SVM	53.22	46.51	51.46	58.73	54.10	51.67	45.42	28.54	39.42
CNN	60.36	57.14	58.56	67.29	68.29	68.87	64.33	80.93	87.34
Inception	66.83	63.03	63.66	72.08	70.32	69.89	71.31	81.36	89.80
PICNN	68.36	66.01	67.79	74.95	71.83	72.90	73.31	f82.67	90.76

We also investigated the recognition rate for each class of phrase in the corpus to better analyze the robustness of our model. In practice, we recorded the recognition rate for all the 101 classes of Chinese phrases and the empty demand using CNN, Inception, PICNN architectures with MFSC, as well as SVM classifier with MWL feature. LDA and RF failed to obtain a classification accuracy above 50% with any of the feature extraction methods so we abandoned these two models in the following experiments. Statistical analysis of the recognition rate for these 101 classes of utterances in the corpus using four classifiers was shown in Table 3.

**Table 3:** Recognition rate analysis for different models.

**Classifier**	**SVM**	**CNN**	**Inception**	**PICNN**
Minimum	0.35	0.59	0.61	0.74
Mean	0.61	0.87	0.89	0.90
Standard Deviation	0.150	0.076	0.071	0.054

From the perspective of each class of demands, we focused on the minimum recognition rate of the above methods. The minimum recognition rate stood for the worst recognition result. It was 74% for PICNN and outperformed the other three models. Our proposed PICNN architecture obtained not only the highest recognition accuracy on the whole dataset but also the highest recognition threshold considering each class of demands.

On the other hand, we also looked into the variance and degree of dispersion in the recognition rates. The standard deviation was 0.054 for PICNN, which showed the least dispersion degree of the recognition rate among classes. It confirmed that the proposed PICNN method had smaller recognition differences among different classes in the corpus and more robust performance.

### 5.2. Recognition differences among classes

To further study the recognition differences among utterances, we analyzed the classification report, summarized the confusion matrix of our PICNN model using the MFSC feature, and recorded the 10 classes of demands which obtained the lowest recognition rate. Detailed information on these demands and how they were misclassified into other classes was illustrated in Table 4.

**Table 4:** Specific information of the 10 classes of demands with the lowest recognition rate.

**Label**	**Misclassified labels (number of samples misclassified)**	**Recognition rate**
38	98 (1), 82 (2), 33 (2), 30 (1), 24 (2), 13 (1), 3 (1), 0 (1)	0.83
77	82 (1), 48 (1), 31 (1), 13 (1), 11 (1), 0 (1)	0.81
79	**85 (14)**, 80 (1), 64 (1), 54 (1), 16 (1)	0.81
85	93 (1), 80 (1), 79 (2), 77 (1), 30 (1)	0.81
92	97 (2), 68 (1), 66 (2), 25 (1), 18(1), 6 (1)	0.81
80	98 (1), 90 (2), 81 (4), 23 (1), 16 (1)	0.79
89	97 (2), 95 (2), 92 (2), 88 (1), 83 (1), 77 (1), 69 (1), 68 (2), 66 (4), 29 (1), 6 (1), 4 (1)	0.79
69	97 (1), 92 (2), 84 (1), 77 (1), 74 (1), 59 (1), 33 (1), 23 (4), 4 (1), 0 (1)	0.76
4	71 (1), 69 (1), 42 (1), 39 (1), 36 (2), 30 (1), 15 (1), 11 (2)	0.75
12	92 (1), 91 (1), 82 (1), 81 (1), 77 (1), 69 (1), 60 (1), 38 (1), 32 (1), 31 (1), 13 (1), 11(1), 5 (1), 3 (2), 1 (1)	0.74

Since we shuffled the whole dataset before model training and testing, there were slight differences in the number of data for each class of demands in the test set. Overall, the number of misclassified samples for each class was no more than six against each false label. However, we still noticed that 14 pieces of sEMG data labeled 79 were classified into label 85. The demand with label 79 was *I want to cut my hair* and the demand with label 85 was *I want to wash my hair*. The phonetic transcriptions of these two utterances in Mandarin were shown in Table 5. They were quite similar to each other considering the contents and pronunciation in Chinese. The differences in muscle activities and sEMG data were not distinguished by feature extraction process and classifier. Meanwhile, we noticed that the demand *I'm a little bit cold* with label 12 obtained the lowest recognition rate using the four classifiers listed above. The phonetic transcription of this utterance was also indicated in Table 5. It was misclassified into another fifteen demands according to the confusion matrix, far exceeding other classes of demands in perspective of misclassification. We would continue to study the reason why this particular demand was difficult to be recognized by the recognition system.

**Table 5:** Phonetic information for utterances labeled 79, 85, and 12.

**Label**	**Demand**	**Mandarin phonetic transcription**	**English translation**
79	我要剪头发	wo3yao4jian3tou2fa4	I want to cut my hair
85	我要洗头发	wo3yao4xi3tou2fa4	I want to wash my hair
12	我有点冷	wo3you3dian3leng3	I'm a little bit cold

### 5.3. Cross-subject experiments and transfer learning

Cross-subject adaption is an essential consideration for sEMG based silent speech recognition system. For the practical application test, we invited four more subjects to take part in the data acquisition process and more experiments were carried out to improve the cross-subject recognition ability of our PICNN model with transfer learning strategies. Data collected from these four subjects (three male subjects and one female subject) was used in this part of the study. The same data collection experiment was conducted, and four pre-trained models, i.e., PICNN, Inception, CNN, and SVM were used for the speech recognition system. We fed the newly collected data into these four models using corresponding features and their model parameters obtained in previous experiments.

A subject-based transfer learning strategy was designed and tested for these four subjects. The core idea of this cross-subject transfer learning was to use part of the new data for model fine-tuning and improve the recognition ability of the pre-trained models. For each subject, we increased the percentage of labeled data used for fine-tuning, from one session to at most eight sessions. The rest of the data was used for model testing.

**Figure 6:**
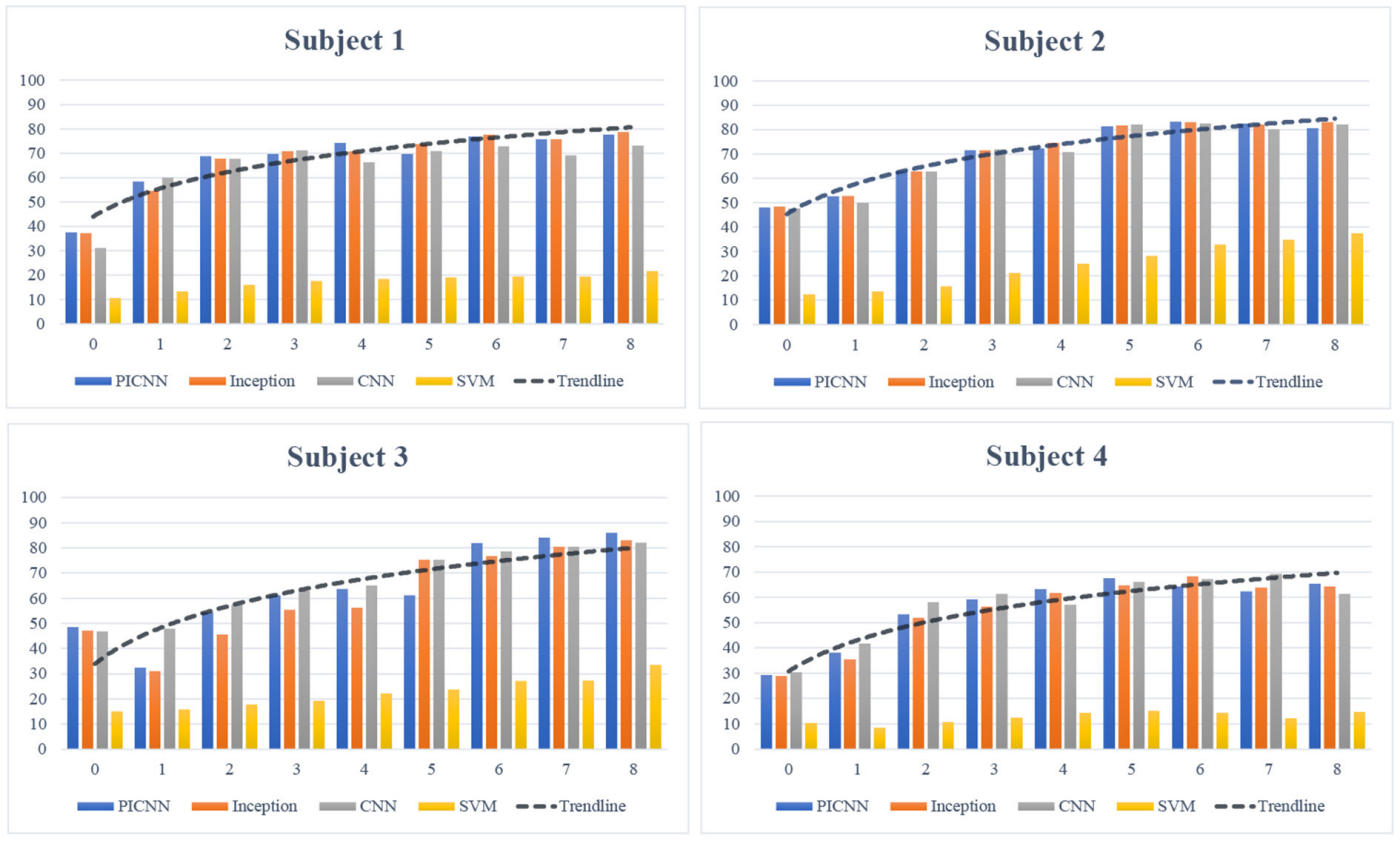
Subject-based transfer learning process. The logarithmic trendline of classification accuracy for each subject was drawn based on the PICNN model.

The rise of classification accuracy for each subject during the transfer learning process is shown in Figure 6, and an obvious increase in classification accuracy of sEMG-based silent speech recognition system for each subject can be observed here. Although there was a slight fluctuation of classification accuracy, with more percentage of new data adopted for model fine-tuning, pre-trained models could improve their recognition ability in cross-subject application scenery. From this perspective, we believed that this cross-subject transfer learning strategy was effective for the implementation of the proposed silent speech recognition system in cross-subject tasks. Considering the differences among each subject and each session of collected data, our newly developed PICNN model still obtained satisfactory recognition accuracy with more sessions involved in fine-tuning, giving the best recognition results for Subject 3 and Subject 4 at the end of transfer learning experiments.

## 6. Discussion

### 6.1. Feature extraction method affects recognition performance

Many studies focus on the development of sEMG features and the influence of feature extraction methods in sEMG pattern recognition, not only for speech recognition (Jou et al., 2006) but also for other applications such as gesture recognition (Ma et al., 2021). For example, the spectral and power features were combined to form a new feature vector in Jou et al. (2006), which was later implemented in a range of sEMG-based speech recognition researches like Wand et al., 2014. It is clear that the combination of features from several domains can improve the recognition ability of sEMG based silent speech recognition system. Interestingly, we observe a more complex conclusion on how the feature extraction method affects sEMG pattern recognition in our research.

Overall, spectral features, especially MFCC and MFSC are relatively more suitable for three CNN-based deep learning architectures with the lowest recognition accuracy of 80.93% for MFCC and CNN pair and highest recognition accuracy of 90.76% obtained by our own model PICNN using MFSC. Both time domain features and their combinations with frequency components fail to work very well in recognition experiments with deep learning methods. While originally proposed based on human auditory characteristics, experimental results prove that MFCC and MFSC can be further adopted in sEMG-based silent speech recognition tasks with promising recognition accuracy. However, it is almost the contrary results when spectral features are applied in machine learning algorithms. Instead, time domain features and their combinations lead to better recognition accuracy for LDA, RF, and SVM models. We check our codes and the python packages we use for model construction and find it may be because of the dimensional reshape of the input feature, in another word it is the curse of dimensionality for spectral features.

### 6.2. Speech recognition methods used in sEMG based SSR

Apart from feature extraction approaches, the selection of speech recognition algorithms has a significant impact on the recognition results. Statistical methods such as Hidden Markov Model (HMM) and Gaussian Mixture Model (GMM) have been used for sEMG-based speech recognition for a long time. For example, in phoneme and word classification task (Zhou et al., 2009), an average classification accuracy of over 98% had been achieved by the proposed GMM classifier for up to 20 words over six subjects. They were efficient recognizers for continuous speech recognition as well (Meltzner et al., 2017). With the development of machine learning algorithms, some popular recognition methods have been adopted to further improve the recognition ability of sEMG-based SSR. Support Vector Machine (SVM), which obtained the best classification accuracy among three machine learning algorithms in our work, also gave a recognition accuracy of over 75% for phoneme classification on 10 subjects (Khan and Jahan, 2018). Linear Discriminant Analysis (LDA) was used for eleven-instruction classification with over 90% classification accuracy (Liu et al., 2020). In recent years, Convolutional Neural Networks have achieved much success in various research fields. Different from the classification of words or phonemes with limited classes, CNN architecture shows satisfactory recognition ability in our work, with at least 80.93% classification accuracy using spectral features MFCC and MFSC on the 100-class Chinese phrases. It is a challenging task to distinguish the right label for the corresponding utterance as there are similar pronunciation and similar contents among these phrases, and the experimental results also confirm that spatial feature extraction using convolutional layers based on spectral feature input can give a much better recognition performance than machine learning algorithms like SVM, RF, and LDA. Furthermore, the proposed PICNN architecture obtains the combination of single-channel spatial feature and cross-channel spatial feature, which is proved to be more effective than previous and commonly used CNN architectures. We believe the parallel inception convolutional module is a valuable spatial feature extractor. It can be cooperated with temporal neural networks to improve speech recognition ability and implemented in more complex applications in future work.

### 6.3. Cross-subject issue in practice

In a review paper on sEMG signal processing, classification, and application, the author pointed out the substantial differences in EMG signal patterns caused by physiological and behavioral differences among users (Phinyomark et al., 2020). The changes in sEMG signals across users brought challenges and discussions for the implementation of EMG signals, not only in silent speech recognition but also in other research fields related to muscle movements (Di Nardo et al., 2017; Hill et al., 2018). Although the subject-based transfer learning could improve the recognition accuracy, the final results differed with the use of sEMG data obtained from different subjects. As demonstrated in Figure 6, for each recognition method, sEMG data collected from Subject 2 and Subject 3 showed relatively higher recognition performance, compared to data collected from the other two subjects. The cross-subject issue in practical applications urges the development of a robust recognition model for sEMG based silent speech recognition system, and it is also an essential consideration when we design our PICNN model.

## 7. Conclusion and outlook

In this paper, we proposed a new deep learning architecture, PICNN, with both high recognition accuracy and robustness. On the 100-class dataset we designed and collected, the best recognition accuracy of 90.76% was achieved by PICNN with MFSC features, which was implemented in sEMG-based silent speech recognition for the first time. Furthermore, to improve the cross-subject recognition ability of our system, we applied subject-based transfer learning with the fine-tuning of the pre-trained PICNN model, making our model more robust for different subjects and users. The experimental results confirmed that our proposed PICNN held promising ability in sEMG-based silent speech recognition tasks, and the silent speech recognition system designed in this paper had great potential for practical application for a wide range of users, including certain patients and the disabled in need.

To better improve the recognition performance of our silent speech recognition system, we would invite more subjects of different ages and health conditions to participate in the data collection process. Considering the target users and application scenarios, we believe the diversity in training and testing data is the essential part of further implementation.

Furthermore, the differences in recognition rates for different demands in the dataset will be learned in future work. We notice that some demands have unsatisfactory recognition rates even when we try different feature extraction methods and classifiers in our experiments. We would like to carry on more experiments with machine learning algorithms and physiological principles of speech activity aspects, with more subjects to find out why these particular demands cannot be recognized. It is expected to further improve the robustness of our system.

## Data availability statement

The raw data supporting the conclusions of this article will be made available by the authors, without undue reservation.

## Ethics statement

The studies involving human participants were reviewed and approved by Institutional Review Board of Tianjin University. The patients/participants provided their written informed consent to participate in this study. Written informed consent was obtained from the individual(s) for the publication of any potentially identifiable images or data included in this article.

## Author contributions

XA, EY, and DM contributed to the conception and design of the study. YZ, LX, and YY organized the database. JW performed the statistical analysis and wrote the first draft of the manuscript. YZ, XZ, and SL wrote sections of the manuscript. All authors contributed to manuscript revision, read, and approved the submitted version.

## Funding

This work was supported in part by grants from the National Natural Science Foundation of China under Grant Nos. 62076250, 61703407, and 61901505.

## Conflict of interest

The authors declare that the research was conducted in the absence of any commercial or financial relationships that could be construed as a potential conflict of interest.

## Publisher's note

All claims expressed in this article are solely those of the authors and do not necessarily represent those of their affiliated organizations, or those of the publisher, the editors and the reviewers. Any product that may be evaluated in this article, or claim that may be made by its manufacturer, is not guaranteed or endorsed by the publisher.

## References

[B1] Atzori M. Cognolato M. Müller H. (2016). Deep learning with convolutional neural networks applied to electromyography data: a resource for the classification of movements for prosthetic hands. Front Neurorobot. 10, 9. 10.3389/fnbot.2016.00009 27656140 PMC5013051

[B2] Bahl L. R. Jelinek F. Mercer R. L. (1983). A maximum likelihood approach to continuous speech recognition. IEEE Trans. Pattern Anal. Mach. Intell. PAMI-5, 179–190. 10.1109/TPAMI.1983.4767370 21869099

[B3] Cai J. Denby B. Roussel-Ragot P. Dreyfus G. Crevier-Buchman L. (2011). “Recognition and real time performances of a lightweight ultrasound based silent speech interface employing a language model,” in Interspeech (Florence), 1005–1008.

[B4] Chollet F. (2017). “Xception: deep learning with depthwise separable convolutions,” in Proceedings of the IEEE Conference on Computer Vision and Pattern Recognition (Honolulu, HI: IEEE), 1251–1258.

[B5] Chowdhury R. H. Reaz M. B. Ali M. A. B. M. Bakar A. A. Chellappan K. Chang T. G. (2013a). Surface electromyography signal processing and classification techniques. Sensors 13, 12431–12466. 10.3390/s130912431 24048337 PMC3821366

[B6] Chowdhury S. K. Nimbarte A. D. Jaridi M. Creese R. C. (2013b). Discrete wavelet transform analysis of surface electromyography for the fatigue assessment of neck and shoulder muscles. J. Electromyogr. Kinesiol. 23, 995–1003. 10.1016/j.jelekin.2013.05.001 23787059

[B7] Chu K. Collins L. Mainsah B. (2020). “Using automatic speech recognition and speech synthesis to improve the intelligibility of cochlear implant users in reverberant listening environments,” in ICASSP 2020 -2020 IEEE International Conference on Acoustics, Speech and Signal Processing (ICASSP) (Barcelona: IEEE), 6929–6933. 10.1109/icassp40776.2020.9054450 PMC7568341 33078056

[B8] Denby B. Schultz T. Honda K. Hueber T. Gilbert J. M. Brumberg J. S. (2010). Silent speech interfaces. Speech Commun. 52, 270–287. 10.1016/j.specom.2009.08.002

[B9] Di Nardo F. Mengarelli A. Strazza A. Agostini V. Knaflitz M. Burattini L.. (2017). A new parameter for quantifying the variability of surface electromyographic signals during gait: the occurrence frequency. J. Electromyogr. Kinesiol. 36, 25–33. 10.1016/j.jelekin.2017.06.006 28688293

[B10] Dobrucki A. B. Pruchnicki P. Plaskota P. Staroniewicz P. Brachmansk S. (2016). Silent speech recognition by surface electromyography. New Trends Dev. Metrol. 81, 145–156. 10.5772/6046733181497

[B11] Englehart K. Hudgins B. Parker P. A. Stevenson M. (1999). Classification of the myoelectric signal using time-frequency based representations. Med. Eng. Phys. 21, 431–438. 10.1016/S1350-4533(99)00066-1 10624739

[B12] Green P. Carmichael J. Hatzis A. Enderby P. Hawley M. Parker M. (2003). “Automatic speech recognition with sparse training data for dysarthric speakers,” in Eighth European Conference on Speech Communication and Technology (Geneva).

[B13] Hill E. C. Housh T. J. Smith C. M. Schmidt R. J. Johnson G. O. (2018). Gender-and muscle-specific responses during fatiguing exercise. J. Strength Condit. Res. 32, 1471–1478. 10.1519/JSC.0000000000001996 29334581

[B14] Hinton G. Deng L. Yu D. Dahl G. E. Mohamed A.-,r. Jaitly N.. (2012). Deep neural networks for acoustic modeling in speech recognition: The shared views of four research groups. IEEE Signal Process Mag. 29, 82–97. 10.1109/MSP.2012.2205597

[B15] Hofe R. Ell S. R. Fagan M. J. Gilbert J. M. Green P. D. Moore R. K.. (2013). Small-vocabulary speech recognition using a silent speech interface based on magnetic sensing. Speech Commun. 55, 22–32. 10.1016/j.specom.2012.02.001

[B16] Hudgins B. Parker P. Scott R. N. (1993). A new strategy for multifunction myoelectric control. IEEE Trans. Biomed. Eng. 40, 82–94. 10.1109/10.204774 8468080

[B17] Hueber T. Chollet G. Denby B. Dreyfus G. Stone M. (2008). “Phone recognition from ultrasound and optical video sequences for a silent speech interface,” in Ninth Annual Conference of the International Speech Communication Association (Brisbane).

[B18] Ioffe S. Szegedy C. (2015). “Batch normalization: accelerating deep network training by reducing internal covariate shift,” in International Conference on Machine Learning (Lille: PMLR), 448–456.

[B19] Ji Y. Liu L. Wang H. Liu Z. Niu Z. Denby B. (2018). Updating the silent speech challenge benchmark with deep learning. Speech Commun. 98, 42–50. 10.1016/j.specom.2018.02.002

[B20] Jong N. S. Phukpattaranont P. (2019). A speech recognition system based on electromyography for the rehabilitation of dysarthric patients: a thai syllable study. Biocybern. Biomed. Eng. 39, 234–245. 10.1016/j.bbe.2018.11.010

[B21] Jou S.-C. Schultz T. Walliczek M. Kraft F. Waibel A. (2006). “Towards continuous speech recognition using surface electromyography,” in Ninth International Conference on Spoken Language Processing (Pittsburgh, PA).

[B22] Kapur A. Kapur S. Maes P. (2018). “Alterego: a personalized wearable silent speech interface,” in 23rd International Conference on Intelligent User Interfaces (Tokyo), 43–53. 10.1145/3172944.3172977

[B23] Karlsson S. Yu J. Akay M. (1999). Enhancement of spectral analysis of myoelectric signals during static contractions using wavelet methods. IEEE Trans. Biomed. Eng. 46, 670–684. 10.1109/10.764944 10356874

[B24] Khan M. Jahan M. (2018). Classification of myoelectric signal for sub-vocal hindi phoneme speech recognition. J. Intell. Fuzzy Syst. 35, 5585–5592. 10.3233/JIFS-161067

[B25] Kim M. Cao B. Mau T. Wang J. (2017). Speaker-independent silent speech recognition from flesh-point articulatory movements using an lstm neural network. IEEE/ACM Trans. Audio Speech Lang. Process. 25, 2323–2336. 10.1109/TASLP.2017.2758999 30271809 PMC6154510

[B26] Kubo T. Yoshida M. Hattori T. Ikeda K. (2013). “Shift invariant feature extraction for semg-based speech recognition with electrode grid,” in 2013 35th Annual International Conference of the IEEE Engineering in Medicine and Biology Society (EMBC) (Osaka: IEEE), 5797–5800. 10.1109/EMBC.2013.6610869 24111056

[B27] Liu H. Dong W. Li Y. Li F. Geng J. Zhu M.. (2020). An epidermal semg tattoo-like patch as a new human-machine interface for patients with loss of voice. Microsyst. Nanoeng. 6, 1–13. 10.1038/s41378-019-0127-5 34567631 PMC8433406

[B28] Liu Z. Wu Z. Li T. Li J. Shen C. (2018). Gmm and cnn hybrid method for short utterance speaker recognition. IEEE Trans. Ind. Inform. 14, 3244–3252. 10.1109/TII.2018.2799928

[B29] Lyu M. Xiong C. Zhang Q. (2014). “Electromyography (emg)-based chinese voice command recognition,” in 2014 IEEE International Conference on Information and Automation (ICIA) (Hailar: IEEE), 926–931.

[B30] Ma C. Guo W. Zhang H. Samuel O. W. Ji X. Xu L.. (2021). A novel and efficient feature extraction method for deep learning based continuous estimation. IEEE Rob. Autom. Lett. 6, 7341–7348. 10.1109/LRA.2021.3097257

[B31] Maslow A. H. (1943). A theory of human motivation. Psychol. Rev. 50, 370. 10.1037/h0054346

[B32] Meltzner G. S. Colby G. Deng Y. Heaton J. T. (2011). “Signal acquisition and processing techniques for semg based silent speech recognition,” in 2011 Annual International Conference of the IEEE Engineering in Medicine and Biology Society (Boston, MA: IEEE), 4848–4851. 10.1109/IEMBS.2011.6091201 22255424

[B33] Meltzner G. S. Heaton J. T. Deng Y. De Luca G. Roy S. H. Kline J. C. (2017). Silent speech recognition as an alternative communication device for persons with laryngectomy. IEEE/ACM Trans. Audio Speech Lang. Process. 25, 2386–2398. 10.1109/TASLP.2017.2740000 29552581 PMC5851476

[B34] Meltzner G. S. Heaton J. T. Deng Y. De Luca G. Roy S. H. Kline J. C. (2018). Development of semg sensors and algorithms for silent speech recognition. J. Neural Eng. 15, 046031. 10.1088/1741-2552/aac965 29855428 PMC6168082

[B35] Mendes Junior J. J. A. Freitas M. L. B. Campos D. P. Farinelli F. A. Stevan S. L. Pichorim S. F. (2020). Analysis of influence of segmentation, features, and classification in semg processing: a case study of recognition of brazilian sign language alphabet. Sensors 20, 4359. 10.3390/s20164359 32764286 PMC7471999

[B36] Mohamed A.-,r. (2014). Deep Neural Network Acoustic Models for ASR (Ph.D. thesis). University of Toronto.

[B37] Morse M. S. Day S. H. Trull B. Morse H. (1989). “Use of myoelectric signals to recognize speech,” in Images of the Twenty-First Century. Proceedings of the Annual International Engineering in Medicine and Biology Society (Seattle, WA: IEEE), 1793–1794.

[B38] Oskoei M. A. Hu H. (2007). Myoelectric control systems–a survey. Biomed. Signal Process. Control 2, 275–294. 10.1016/j.bspc.2007.07.009

[B39] Phinyomark A. Campbell E. Scheme E. (2020). “Surface electromyography (emg) signal processing, classification, and practical considerations,” in Biomedical Signal Processing (Singapore: Springer), 3–29.

[B40] Phinyomark A. Limsakul C. Phukpattaranont P. (2009). A novel feature extraction for robust emg pattern recognition. arXiv preprint arXiv:0912.3973. 10.48550/arXiv.0912.3973

[B41] Phinyomark A. Phukpattaranont P. Limsakul C. (2012). Feature reduction and selection for emg signal classification. Expert. Syst. Appl. 39, 7420–7431. 10.1016/j.eswa.2012.01.102

[B42] Rameau A. (2020). Pilot study for a novel and personalized voice restoration device for patients with laryngectomy. Head Neck 42, 839–845. 10.1002/hed.26057 31876090

[B43] Rashno E. Akbari A. Nasersharif B. (2019). A “convolutional neural network model based on neutrosophy for noisy speech recognition,” in *2019 4th International Conference on Pattern Recognition and Image Analysis (IPRIA)* (Tehran: IEEE), 87–92.

[B44] Samuel O. W. Zhou H. Li X. Wang H. Zhang H. Sangaiah A. K.. (2018). Pattern recognition of electromyography signals based on novel time domain features for amputees' limb motion classification. Comput. Electr. Eng. 67, 646–655. 10.1016/j.compeleceng.2017.04.003

[B45] Schultz T. Wand M. Hueber T. Krusienski D. J. Herff C. Brumberg J. S. (2017). Biosignal-based spoken communication: a survey. IEEE/ACM Trans. Audio Speech Lang. Process. 25, 2257–2271. 10.1109/TASLP.2017.2752365

[B46] Simonyan K. Zisserman A. (2014). Very deep convolutional networks for large-scale image recognition. arXiv preprint arXiv:1409.1556. 10.48550/arXiv.1409.1556

[B47] Smith L. H. Hargrove L. J. Lock B. A. Kuiken T. A. (2011). Determining the optimal window length for pattern recognition-based myoelectric control: balancing the competing effects of classification error and controller delay. IEEE Trans. Neural Syst. Rehabil. Eng. 19, 186–192. 10.1109/TNSRE.2010.2100828 21193383 PMC4241762

[B48] Srisuwan N. Phukpattaranont P. Limsakul C. (2018). Comparison of feature evaluation criteria for speech recognition based on electromyography. Med. Biol. Engi. Comput. 56, 1041–1051. 10.1007/s11517-017-1723-x 29134413

[B49] Stone S. Birkholz P. (2016). “Silent-speech command word recognition using electro-optical stomatography,” in Interspeech (San Francisco, CA), 2350–2351.

[B50] Szegedy C. Ioffe S. Vanhoucke V. Alemi A. A. (2017). “Inception-v4, inception-resnet and the impact of residual connections on learning,” in Thirty-First AAAI Conference on Artificial Intelligence (San Francisco, CA).

[B51] Szegedy C. Liu W. Jia Y. Sermanet P. Reed S. Anguelov D.. (2015). “Going deeper with convolutions,” in Proceedings of the IEEE Conference on Computer Vision and Pattern Recognition (Boston, MA: IEEE), 1–9.

[B52] Szegedy C. Vanhoucke V. Ioffe S. Shlens J. Wojna Z. (2016). “Rethinking the inception architecture for computer vision,” in Proceedings of the IEEE Conference on Computer Vision and Pattern Recognition (Las Vegas, NV: IEEE), 2818–2826.

[B53] Tkach D. Huang H. Kuiken T. A. (2010). Study of stability of time-domain features for electromyographic pattern recognition. J. Neuroeng. Rehabil. 7, 1–13. 10.1186/1743-0003-7-21 20492713 PMC2881049

[B54] Wand M. Janke M. Schultz T. (2014). Tackling speaking mode varieties in emg-based speech recognition. IEEE Trans. Biomed. Eng. 61, 2515–2526. 10.1109/TBME.2014.2319000 24760900

[B55] Wu J. Zhao T. Zhang Y. Xie L. Yan Y. Yin E. (2021). “Parallel-inception cnn approach for facial semg based silent speech recognition,” in 2021 43rd Annual International Conference of the IEEE Engineering in Medicine Biology Society (EMBC) (Mexico: IEEE), 554–557. 10.1109/EMBC46164.2021.9630373 34891354

[B56] Xie H. Wang Z. (2006). Mean frequency derived via hilbert-huang transform with application to fatigue emg signal analysis. Comput. Methods Programs Biomed. 82, 114–120. 10.1016/j.cmpb.2006.02.009 16616796

[B57] Xiong W. Wu L. Alleva F. Droppo J. Huang X. Stolcke A. (2018). “The microsoft 2017 conversational speech recognition system,” in 2018 IEEE International Conference on Acoustics, Speech and Signal Processing (ICASSP) (Calgary, AB: IEEE), 5934–5938.

[B58] Yang R. Cheng G. Miao H. Li T. Zhang P. Yan Y. (2021). Keyword search using attention-based end-to-end asr and frame-synchronous phoneme alignments. IEEE/ACM Trans. Audio Speech Lang. Process. 29, 3202–3215. 10.1109/TASLP.2021.3120632

[B59] Yu D. Deng L. (2016). Automatic Speech Recognition. London: Springer.

[B60] Zeghidour N. Usunier N. Kokkinos I. Schaiz T. Synnaeve G. Dupoux E. (2018). “Learning filterbanks from raw speech for phone recognition,” in 2018 IEEE International Conference on Acoustics, Speech and Signal Processing (ICASSP) (Calgary, AB: IEEE), 5509–5513.

[B61] Zhang M. Wang Y. Zhang W. Yang M. Luo Z. Li G. (2020). Inductive conformal prediction for silent speech recognition. J. Neural Eng. 17, 066019. 10.1088/1741-2552/ab7ba0 32120355

[B62] Zheng F. Zhang G. Song Z. (2001). Comparison of different implementations of mfcc. J. Comput. Sci. Technol. 16, 582–589. 10.1007/BF02943243

[B63] Zhou Q. Jiang N. Hudgins B. (2009). Improved phoneme-based myoelectric speech recognition. IEEE Trans. Biomed. Eng. 56, 2016–2023. 10.1109/TBME.2009.2024079 19535319

